# In Situ Revascularization with a Rifampicin-Soaked Prosthesis to Treat Bare Iliac Artery Stent Infection: A Case Report

**DOI:** 10.3400/avd.cr.21-00052

**Published:** 2021-09-25

**Authors:** Munetaka Hashimoto, Yoshihisa Tamate, Hiroko Sato, Akihiko Murakami, Shunsuke Shibuya, Naoki Yanagawa

**Affiliations:** 1 Department of Vascular Surgery, Iwate Prefectural Isawa Hospital, Oshu, Iwate, Japan; 2 Department of Molecular Diagnostic Pathology, Iwate Medical University School of Medicine, Morioka, Iwate, Japan

**Keywords:** bare stent infection, infected aneurysm, rifampicin-soaked graft

## Abstract

Bare stent infection is an extremely rare complication of endovascular treatment. In such cases, surgical resection of the infected bare stent and revascularization are recommended; however, the revascularization strategy remains controversial. We present a case of a 78-year-old man with an infected aneurysm caused by a bare iliac artery stent infection. We resected the infected aneurysm and performed in situ anatomic reconstruction using a rifampicin-soaked prosthesis with omental coverage. The patient had no reinfection at the 3-year follow-up. Therefore, this procedure may be a useful treatment for bare iliac artery stent infections.

## Introduction

Recently, endovascular treatment (EVT) for peripheral arterial disease has shown remarkable developments. The use of iliac stenting has become widespread because the procedure is minimally invasive and achieves long-term patency. Bare stent infection—which causes fever, chills, local pain, malaise, and leukocytosis—is a rare complication of EVT. Only a few cases have been reported to date, of which half developed within a month post-EVT.^1,2)^

Conservative treatment with antibiotics alone has poor results, and some cases may be fatal due to severe sepsis or rupture of infected aneurysm; therefore, surgical debridement and revascularization are required.^3,4)^ Extra-anatomical revascularization had been performed in most previous studies. However, only a few reports mentioned the use of in situ anatomical reconstruction using autologous vein grafts or allografts.^1,2,5)^ Several reports have suggested that in situ anatomical revascularization with a rifampicin-soaked prosthesis may be useful for treating aortic graft infections. However, to our knowledge, there is no report on the use of a rifampicin-soaked prostheses for in situ revascularization in patients with iliac stent infections.^6–8)^ Here, we report a rare case of a patient who had an infected aneurysm caused by a bare iliac artery stent infection. The patient was treated by surgical debridement followed by in situ anatomical revascularization using a rifampicin-soaked prosthesis with pedicled omental coverage. At the 3-year follow-up, the patient was alive and well with no recurrence of graft infection.

Informed consent was obtained from the patient to publish his details. The research ethics committee of Iwate Prefectural Isawa Hospital provided approval for the use of a rifampicin graft in this case and for publication of this case report (approval No. 2021-02).

## Case Report

A 78-year-old man with a history of hypertension and benign prostatic hyperplasia, and no history of any infectious diseases, underwent endovascular stenting (S.M.A.R.T. Control C10060SL, Cordis, Santa Clara, CA, USA) at another hospital for chronic occlusion of the left iliac artery, which had been causing intermittent claudication. No prophylactic antibiotics were administered to the patient during the EVT procedure. The patient was discharged the next day without any complications. However, he developed fever and back pain 20 days later. Contrast-enhanced computed tomography (CT) was suggestive of an infected aneurysm at the site of the left iliac artery stent, and the patient was referred to our hospital.

**Figure 1:**
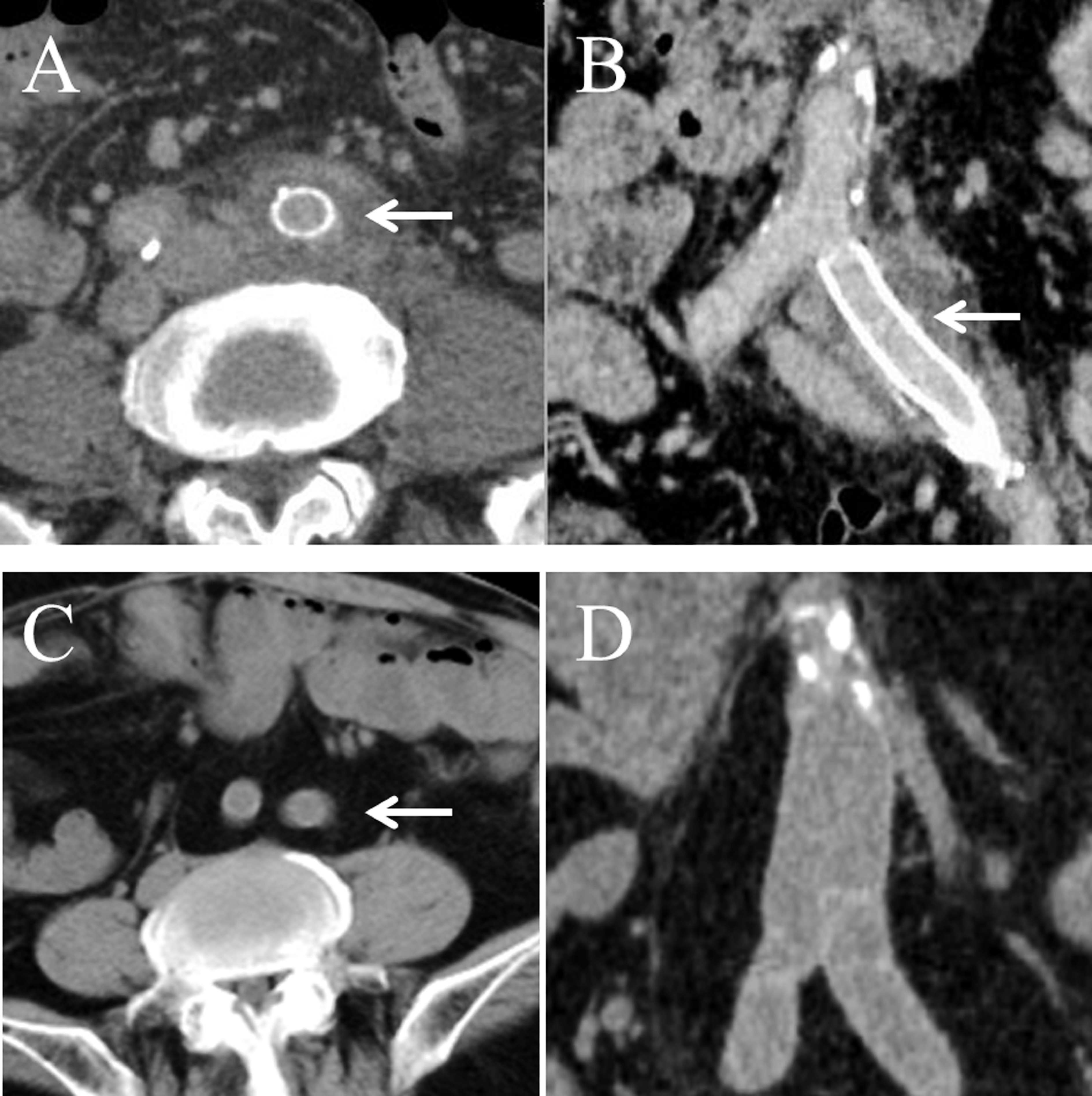
Fig. 1 (**A**)(**B**) Preoperative computed tomography (CT) images showing the bare iliac stent (arrows) and infected aneurysm. (**C**)(**D**) Postoperative CT images at the 2-year follow-up showing the prosthesis graft with no reinfection. The graft was wrapped by the omental flap (arrow), which is seen as a low density around the graft.

The patient was 160 cm tall, weighed 62.8 kg, and had a blood pressure of 156/80 mmHg, a pulse of 66 beats/min, and a body temperature of 38°C. Blood tests revealed the following: white blood cells, 13130/µl; red blood cells, 3.33 million/µl; hemoglobin, 9.9 g/dl; platelet count, 190,000/µl; blood urea nitrogen, 26.7 mg/dl; creatinine, 0.93 mg/dl; albumin, 2.4 g/dl; and C-reactive protein, 24.76 mg/dl. CT findings revealed thickening of the arterial wall and an aneurysm of the left common iliac artery at the site of the stent (**Figs. 1A** and **1B**). On the basis of these findings, he was diagnosed with an infected aneurysm due to bare stent infection and underwent surgical treatment.

**Figure 2:**
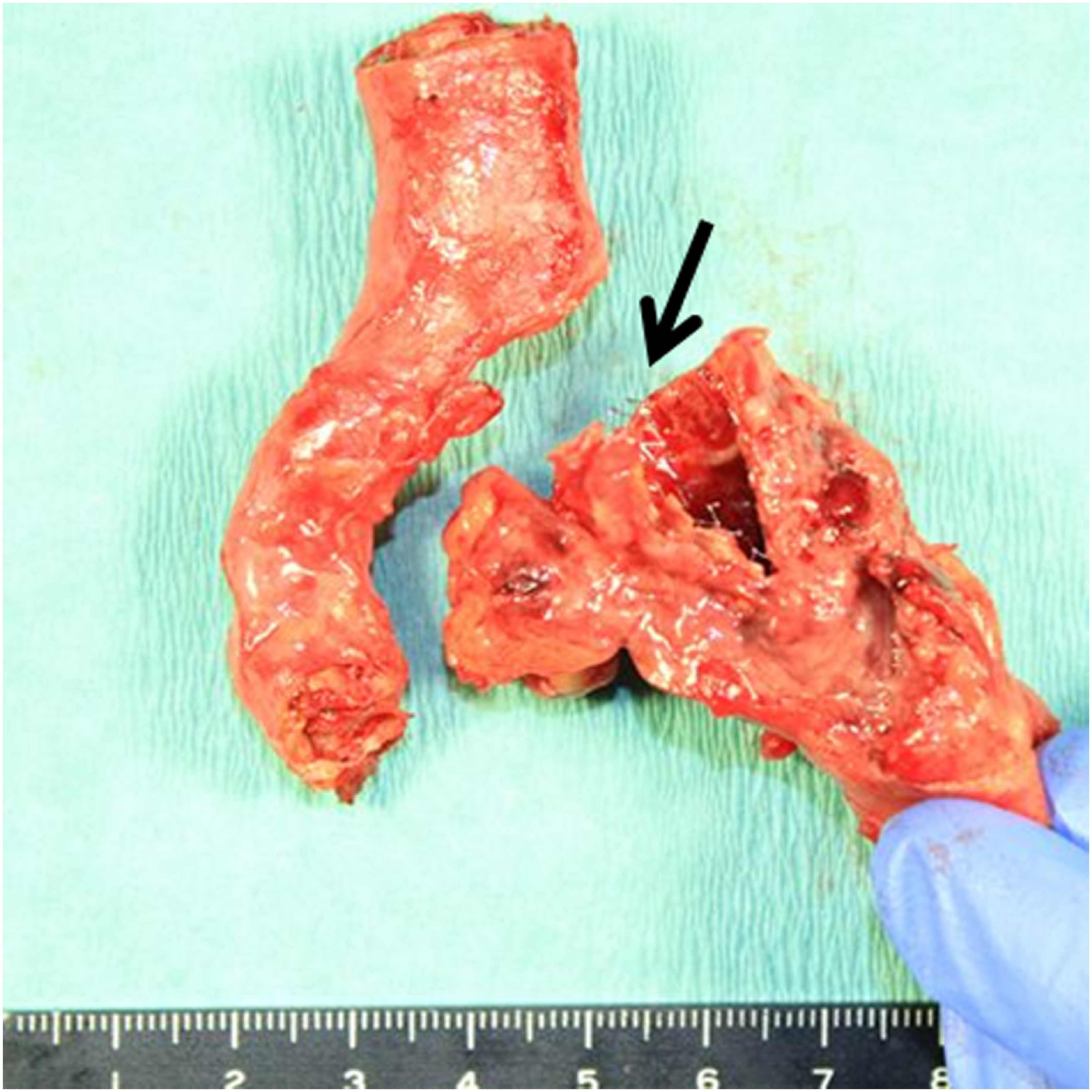
Fig. 2 The resected specimen showing the terminal aorta and bilateral iliac arteries. The bare stent (arrow) is seen in an aneurysm of the left common iliac artery.

The terminal abdominal aorta and bilateral common iliac arteries were removed because the infection had extended to the aortic bifurcation (**Fig. 2**). The surrounding infected tissue was debrided, and the terminal aorta was replaced with a rifampicin-soaked bifurcated prosthesis. A pedicled omental flap based on the left gastroepiploic artery was then guided in a retrocolic fashion into the retroperitoneal cavity, to wrap the graft around its entire length.

The rifampicin-soaked prosthesis was prepared as follows. First, 300-mg rifampicin capsules (Sandoz Corporation, Tokyo, Japan) were dissolved in a mixture of 6-ml high-pressure sterilized polyoxyethylene sorbitan monooleate (Tween 80, Wako Seiyaku, Tokyo, Japan) and 54-ml distilled water. This solution was then sterilized in a Millex-HV Syringe Filter Unit (0.45 µm polyvinylidene difluoride, Merck Millipore, Carrigtwohill, County Cork, Ireland). Finally, an 18×10-mm prosthesis (Hemashield Platinum, Maquet, San Jose, CA, USA) was soaked in this rifampicin solution for 15 min.

**Figure 3:**
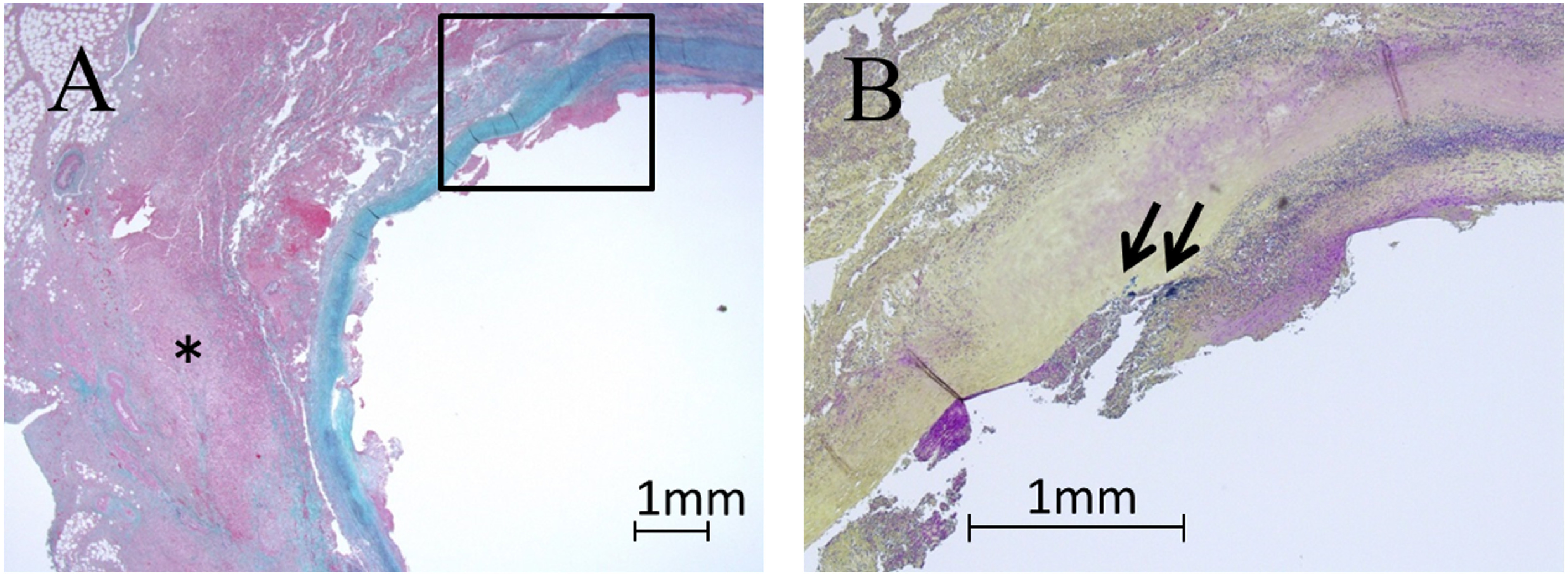
Fig. 3 The histopathological images are shown. (**A**) Elastica–Masson stain (×20): many inflammatory cells are seen infiltrating the intima, media, and adventitia. Abscesses are seen in the adventitial layer (*). (**B**) Gram stain (×100) of the square part in (**A**). Masses of Gram-positive cocci (arrows) are seen in the intima and media.

Histopathology of the surgically excised artery specimen revealed many inflammatory cells infiltrating the intima, media, and adventitia. Abscesses and masses of Gram-positive cocci were found in all arterial layers, suggestive of an infected aneurysm (**Fig. 3**).

*Staphylococcus aureus* was detected by a bacterial culture of the surgical specimen. Based on the results of his antibiotic susceptibility test, the patient was administered tazobactam/piperacillin 13.5 g/day intravenously for 4 weeks, followed by amoxicillin 750 mg/day orally. However, oral medication was discontinued after 2 weeks due to a side effect, i.e., diarrhea.

Two postoperative complications were also treated. For adhesive bowel obstruction, partial small bowel resection was performed. For aspiration pneumonia, meropenem hydrate 1.5 g/day was administered for 1 week.

The patient was discharged and fully recovered from his illness. The patient still visits our hospital every 3–6 months for blood tests, Doppler ultrasonography, and CT. Postoperative CT images at the 2-year follow-up revealed no signs of reinfection (**Figs. 1C** and **1D**). At the 3-year follow-up, the graft remained patent, and no complications were observed.

## Discussion

Conservative treatment for bare stent infections has poor outcomes and may be fatal if there is severe sepsis or rupture of the infected aneurysm. Therefore, early aggressive surgical debridement and revascularization are recommended.^1,2,5)^

Extra-anatomical revascularization, which had been performed in most previous studies, is considered to have a low risk of local reinfection because a new graft is not placed at the infected site. However, it has a low rate of graft patency, a high rate of lower extremity amputation, and poor postoperative prognosis. In addition, blowout of the sutured and closed aortic ends is a devastating potential complication.^1–3,5)^

Several recent studies have documented the usefulness of in situ revascularization for the treatment of aortic graft infections.^7,8)^ Furthermore, a meta-analysis of treatments for aortic graft infections documented that in situ anatomical revascularization has lower estimated event rates for limb amputation, conduit failure, and mortality compared to extra-anatomical revascularization.^7)^ Therefore, we decided to treat our patient with in situ revascularization.

Autologous veins, fresh or frozen allografts, or silver or antibiotic-bonded prostheses may be used as grafts for anatomic reconstruction.^6,7)^ In Japan, there is a limited variety of available grafts because allografts and silver-bonded grafts are not readily available.

Autologous vein grafts are commonly prepared from the great saphenous vein or the femoral vein. Although autologous vein grafts have a low risk of infection, they are associated with other disadvantages.^3,7)^ First, since a vessel diameter of ≥6 mm is recommended, small veins cannot be used. Second, their use is contraindicated in patients with a history of deep vein thrombosis. Third, there is a risk of a substantial size mismatch, which may be resolved by combining veins to make a larger graft.^9)^ However, since it takes time to harvest and combine these grafts, the operative time substantially increases, which is a great concern for fragile patients and those in life-threatening situations. There are also risks associated with harvesting, such as infection of the harvested vein wound, leg swelling, venous stasis, and chronic venous hypertension.^3,7)^

Rifampicin-soaked prostheses are adapted to the aortic diameter and do not take time to prepare. Furthermore, fabric grafts are readily available, and rifampicin solutions may be easily prepared in hospitals.

The use of a vein graft is preferable to reduce the risk of reinfection. However, in this case, a rifampicin-soaked prosthesis was used because the patient’s saphenous and femoral veins were narrow and inadequate for grafting. In addition, as this patient was elderly, the operation time had to be reduced as far as possible. Because it is difficult to obtain a highly concentrated rifampicin solution in Japan, most facilities dissolve rifampicin powder to make a 0.1%–0.5% solution. For this case, a 0.5% formulation was prepared. Additionally, the prosthesis was completely covered with a pedicled omental flap to prevent reinfection. Neither graft reinfection nor rifampicin-related complications were observed in this patient. We performed in situ revascularization because we believed we had sufficiently removed the infected area and considered the risk of reinfection to be very low. It should be noted that revascularization for the claudication may not necessarily be mandatory if life-saving is a priority.

As several surgical options for treating bare stent infections are available, the most appropriate surgical method and graft should be chosen according to the patient’s condition.^10)^ In situ reconstruction using a rifampicin-soaked prosthesis may be a useful option for patients in whom sufficient autologous veins are not available or for those who are expected to have difficulty tolerating prolonged open surgery.

After the surgery, appropriate antibiotic therapy is necessary. There is no evidence regarding the appropriate duration of antibiotic therapy, but a previous report had suggested that the duration should range from 6 weeks to lifelong.^3)^ The patient in this case received antibiotics for 6 weeks, and although we wanted to continue antibiotic therapy for as long as possible, it was discontinued due to a side effect, i.e., diarrhea. The patient was alive without any recurrence of infection at the 3-year follow-up. However, further follow-ups are required because there is still a risk of developing graft reinfection in the future.

## Conclusion

We reported a case of a patient with an infected iliac artery aneurysm due to bare stent infection, which is an extremely rare complication of EVT. Resection of the infected aneurysm and in situ anatomical reconstruction using a rifampicin-soaked prosthesis with pedicled omental coverage may be a useful treatment for bare stent infection.

## References

[1] Bosman WM, Borger van der Burg BL, Schuttevaer HM, et al. Infections of intravascular bare metal stents: a case report and review of literature. Eur J Vasc Endovasc Surg 2014; 47: 87-99. 24239103 10.1016/j.ejvs.2013.10.006

[2] Hogg ME, Peterson BG, Pearce WH, et al. Bare metal stent infections: case report and review of the literature. J Vasc Surg 2007; 46: 813-20. 17903662 10.1016/j.jvs.2007.05.043

[3] Kilic A, Arnaoutakis DJ, Reifsnyder T, et al. Management of infected vascular grafts. Vasc Med 2016; 21: 53-60. 26584886 10.1177/1358863X15612574

[4] Myles O, Thomas WJ, Daniels JT, et al. Infected endovascular stents managed with medical therapy alone. Catheter Cardiovasc Interv 2000; 51: 471-6. 11108684 10.1002/1522-726x(200012)51:4<471::aid-ccd21>3.0.co;2-t

[5] Quintas A, Alves G, Aragão de Morais J, et al. Iliac artery reconstruction with femoral vein after bare metal stent infection. EJVES Short Rep 2017; 34: 28-31. 28856330 10.1016/j.ejvssr.2017.01.001 PMC5576156

[6] Oderich GS, Bower TC, Hofer J, et al. In situ rifampin-soaked grafts with omental coverage and antibiotic suppression are durable with low reinfection rates in patients with aortic graft enteric erosion or fistula. J Vasc Surg 2011; 53: 99-107.e7. 21184932 10.1016/j.jvs.2010.08.018

[7] O’Connor S, Andrew P, Batt M, et al. A systematic review and meta-analysis of treatments for aortic graft infection. J Vasc Surg 2006; 44: 38-45.e8. 16828424 10.1016/j.jvs.2006.02.053

[8] Ducasse E, Calisti A, Speziale F, et al. Aortoiliac stent graft infection: current problems and management. Ann Vasc Surg 2004; 18: 521-6. 15534730 10.1007/s10016-004-0075-9

[9] Clagett GP, Bowers BL, Lopez-Viego MA, et al. Creation of a neo-aortoiliac system from lower extremity deep and superficial veins. Ann Surg 1993; 218: 239-48; discussion, 248-9. 8373267 10.1097/00000658-199309000-00003 PMC1242955

[10] Kan CD, Lee HL, Yang YJ. Outcome after endovascular stent graft treatment for mycotic aortic aneurysm: a systematic review. J Vasc Surg 2007; 46: 906-12. 17905558 10.1016/j.jvs.2007.07.025

